# Structure-Informed Protein Language Models are Robust Predictors for Variant Effects

**DOI:** 10.21203/rs.3.rs-3219092/v1

**Published:** 2023-08-03

**Authors:** Yuanfei Sun, Yang Shen

**Affiliations:** 1 Department of Electrical and Computer Engineering, Texas A&M University, College Station, 77843, Texas, USA.; 2 Department of Computer Science and Engineering, Texas A&M University, College Station, 77843, Texas, USA.; 3 Institute of Biosciences and Technology and Department of Translational Medical Sciences, Texas A&M University, Houston, 77030, Texas, USA.

**Keywords:** Variant effect prediction, protein sequences, protein structures, protein language models, multimodal machine learning.

## Abstract

Predicting protein variant effects through machine learning is often challenged by the scarcity of experimentally measured effect labels. Recently, protein language models (pLMs) emerge as zero-shot predictors without the need of effect labels, by modeling the evolutionary distribution of functional protein sequences. However, biological contexts important to variant effects are implicitly modeled and effectively marginalized. By assessing the sequence awareness and the structure awareness of pLMs, we find that their improvements often correlate with better variant effect prediction but their tradeoff can present a barrier as observed in over-finetuning to specific family sequences. We introduce a framework of structure-informed pLMs (SI-pLMs) to inject protein structural contexts purposely and controllably, by extending masked sequence denoising in conventional pLMs to cross-modality denoising. Our SI-pLMs are applicable to revising any sequence-only pLMs through model architecture and training objectives. They do not require structure data as model inputs for variant effect prediction and only use structures as context provider and model regularizer during training. Numerical results over deep mutagenesis scanning benchmarks show that our SI-pLMs, despite relatively compact sizes, are robustly top performers against competing methods including other pLMs, regardless of the target protein family’s evolutionary information content or the tendency to overfitting / over-finetuning. Learned distributions in structural contexts could enhance sequence distributions in predicting variant effects. Ablation studies reveal major contributing factors and analyses of sequence embeddings provide further insights. The data and scripts are available at https://github.com/Stephen2526/Structure-informed_PLM.git.

## 1 Introduction

The workhorse molecule of life, proteins play a central role in cellular functions. Their variations in humans and pathogens often lead to genetic diseases and therapeutic resistance, respectively. The ability to decipher the association between protein variations and resulting effects could facilitate prognostics and therapeutics for diseases. Although multiplexed assays of variant effects (MAVE), such as deep mutational scanning (DMS) and massively parallel reporter assay (MPRA), are increasingly generating data of variant effects ranging from protein stability to cell viability [1, 2], their speed and applicability can be dwarfed by the amount of variants and effects to characterize. Therefore, there is a pressing demand to develop high-throughput and high-accuracy computational tools for variant effect prediction, ideally with mechanistic explainability.

The task of predicting functional effects of genetic variations has been recently pursued by machine learning methods, supervised and unsupervised. Experimental measurements of effects ranging from protein properties to clinical significance have been curated in public databases and provided labels for supervised machine learning. To predict such labels, input features for supervised machine learning models are often manually engineered, such as physicochemical properties of sequence and structure, evolutionary profiles and conservation, or interaction features, as seen in representative methods such as DEOGEN2 [3], PolyPhen-2 [4] and MutPred2 [5]. The “features” can also be representations learned from sequence data using neural networks, as explored in PrimateAI [6], UniRep [7] and TAPE [8].

Compared to supervised predictors, unsupervised ones do not rely on labels that tend to be scarce and rather exploit the abundance of unlabeled sequence data. They often estimate variant effects with evolutionary landscapes inferred from sequence data (especially homologous sequences of the target protein). SIFT [9] initiated this line of work with a first-order or site-independent model using position-specific-scoring-matrix (PSSM). EVmutation [10] extended it by considering pairwise interactions in a 20-state Potts model. DeepSequence [11] further introduced higher-order interactions with a latent variable model (variational autoencoder), and scored variants by sampling multiple times on the ELBO (evidence lower bound) of the log likelihood for the wild type and any given variant sequence. EVE [12] subsequently enhanced the DeepSequence architecture by factorizing decoder weights with the Gaussian distribution. Multiple sequence alignments (MSAs) are required for the unsupervised models described above, which poses challenges to certain cases without sufficient homologous sequences to reveal evolutionary information (such as orphan proteins and designed proteins). WaveNet [13] eliminated this requirement, thus alignment-free, by training an autoregressive model using the task of predicting the next residue.

A recent wave of unsupervised variant effect predictors is instigated by protein language models (pLMs). Unlike aforementioned unsupervised models, they learn the evolutionary information across families of homologous proteins, using large-scale corpora of protein sequences (such as Pfam, UniRef and BFD) and deep-learning advances in natural language processing (such as LSTM and Transformers). UniRep [7] and SeqVec [14] started with LSTM models. TAPE [8] benchmarked ResNet, LSTM, and transformers and showed the superiority of transformers. Since then, a series of transformer-based pLMs have been developed with increasing size and complexity, from 10^8^ to 10^10^ parameters, represented by ESM-1b [15], ProGen [16], ProtTrans [17], MSA-transformer [18], ESM-1v [19] and ESM-2 [20]. Importantly, self-supervised pretraining of these pLMs learned contexualized representations, attentions, and distributions of amino acids from functionally-fit sequences, which is informative in evolutionary, structural and functional contexts and beneficial to various downstream tasks (including variant effect prediction [19, 21, 22], protein function prediction [23, 24], supervised few-shot protein engineering [25], and protein design [16, 26].) A summary of comparisons among these pLMs can be found in Table 6.

Being a state of the art for variant effect prediction, especially in the zero-shot scenario where no labels of variant effects are used, pLMs still have major limitations to overcome [27]. Although variations originate in genetic and protein sequences, their effects are often manifested in biological contexts and may not be adequately captured in a model with sequence information alone. Moreover large-scale pLMs are prone to overfitting to training sequences [19], which hurts the robustness of variant effect predictors.

**Fig. 1:**
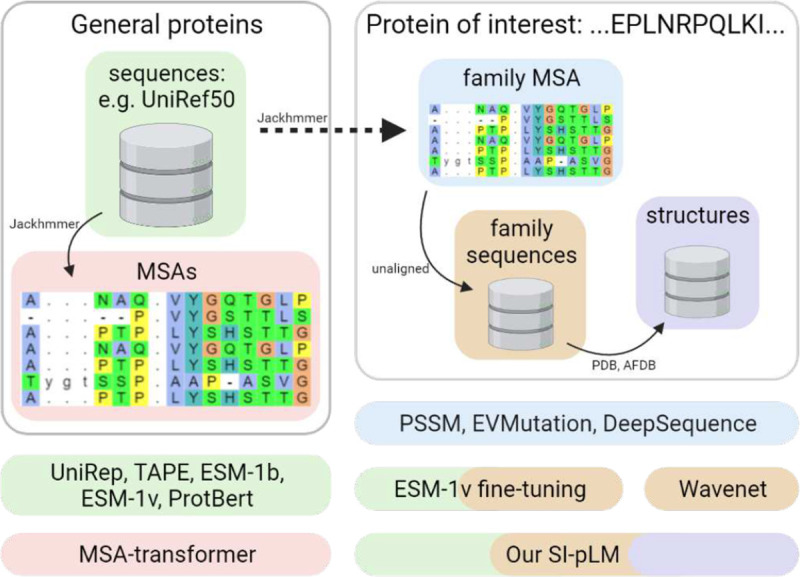
Conceptual differences among competing methods and our structure-informed protein language models (SI-pLMs) from the data perspective. Whereas many family-specific models are trained over aligned (blue) or unaligned sequences (orange) in a protein family, pLMs are often pre-trained over unaligned (green) or aligned sequences (red) in the protein universe and some of them can be fine-tuned over family sequences. In contrast, our SI-pLMs after pretraining are finetuned with both family datasets of sequences and structures.

To address the aforementioned limitations of pLMs for variant effect prediction, we have developed a framework to introduce protein structures, an additional modality to protein sequences, to pLMs as a context provider and model regularizer. Compared to the state-of-the-art pLMs, our models also start with pre-training over representative domain sequences for global albeit coarse-grained evolutionary distributions. But they are structurally informed during fine-tuning over target protein family’s sequences and structures for local and fine-grained evolutionary distributions (Fig. 1).

In developing such a structure-informed pLM, there are a few challenges in data and in machine learning formulation. First, the two modalities of protein data, 1D sequences and 3D structures, are imbalanced. Compared to that of sequences, the amount of experimentally determined structures in the Protein Data Bank is orders of magnitude less. The recent breakthroughs in protein structure prediction (AF2 [28], RoseTTAFold [29] and ESMFold [20]) reduce but do not remove the modality imbalance. Second, variant structures are mostly unavailable (and impossible to enumerate) experimentally; whereas predicting conformational changes upon sequence variation is still challenging to the best structure predictors [30]. These led to the often unavailability of structures as model inputs for variant effect predictors. Last, despite recent works incorporating structures into pLMs through multi-task learning [31], serial encoding [32] and conditioning [33], some of which demand protein structures as part of model inputs, it remains largely unanswered how to rationally introduce structure data into the formulation of masked language modeling in pLMs and how to practically enhance rather than dilute the sequence-derived evolutionary information with structures for the granularity needed for variant effect prediction.

**Fig. 2:**
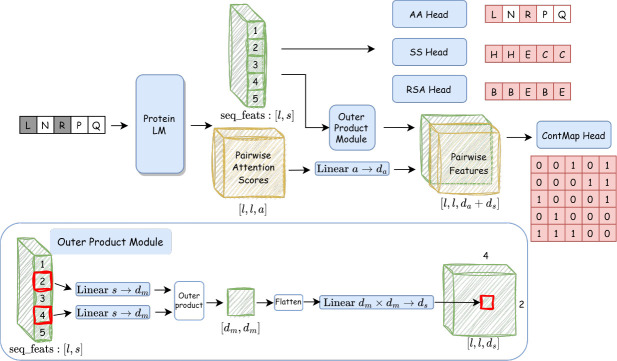
Model architecture of our structure-informed protein language models based on cross-modal sequence and structure denoising. Our model only takes sequential input of noised amino acid types (gray ones are masked), which is easily applicable to variant sequences without structures during inference. Protein LM module is warm-started from a pretrained in-house protein BERT model and generates features for denoising modules. Embeddings after the last layer in pLM, after an outer product moduel, and attention matrices from all heads are concatenated as features for individual amino acids and their pairs, respectively, for denoising sequences and decoding structures. Specifically, the masked amino acids are recovered by amino acid (AA) prediction head. And three structural properties: secondary structure(SS), relative solvent accessibility (RSA) and contact map (ContMap), are classified with their corresponding prediction heads (decoders) in a dense manner.

To address the challenges above, we have introduced the formulation of cross-modality masked modeling with the task decomposition of conventional sequence-only masked modeling and newly introduced sequence-to-structure masked modeling (Sec. 3.2). In this way we reach a flexible framework of SI-pLM where any conventional sequence-only pLMs can be appended with structural decoders in neural network architectures and regularized by self-supervised masked sequence-to-structure tasks (illustrated in Fig. 2 and detailed in Sec. 3.3). With a controllable hyperparameter to adjust the level of injecting structure awareness, this framework is more generic compared to conventional sequence-only pLMs as it reduces to the latter when the hyperparameter is set at zero. Furthermore, during inference, our framework does not require any structure inputs; and during training, it does not require variant structures and can utilize both paired sequence–structure data and unpaired sequences (with structures absent).

Under the framework of SI-pLMs, we first define and evaluate sequence awareness and structure awareness in conventional sequence-only pLMs, which unravels pLMs’ effectiveness in variant effect prediction and motivates our approach to explicitly injecting structure awareness in a controllable fashion (Sec. 2.1). With our curated structures from experiments (PDB) and predictions (AlphaFold DB), we then assessed our SI-pLMs against competing methods over 35 DMS datasets of variant fitness (Sec. 2.3). We found their ranking performances among the best compared to models of 2–12 times more parameters and robust across families of various sequence information (measured by MSA depth). Importantly, we found that fine-tuned family-specific pLMs can perform worse than pre-trained meta pLMs in family-specific variant effect prediction (Sec. 2.2) and such over-finetuning or “overfit” can be mitigated by structural information incorporated in our SI-pLMs (which enhances another sense of robustness). Compared to using paired sequence–structure data only, SI-pLMs performances in variant effect prediction are further improved when unpaired sequences (with structures absent) are additionally considered (Sec. 2.3), regardless of the extent of structure availability in individual families. Learning distributions in structural contexts not only regularized learning sequence distributions but could also improve the latter’s variant scoring capability (Sec. 2.4). Lastly, we reveal the main contributing factors of our SI-pLMs in ablation studies (Sec. 2.5) and assess how SI-pLMs affect the latent embeddings of variant sequences toward variant effect prediction (Sec. 2.6).

## 2 Results

### 2.1 Fitness sensitivity in pLMs is supported by the underlying sequence and structure awareness

Protein sequence modeling, especially through transformer-based protein language models (pLMs), has been shown effective for variant fitness prediction without using fitness labels during training. Trained over protein corpora of natural sequences, pLMs can learn the distribution of amino acid compositions underlying functional sequences and predict variant effects with estimated log odds (the ratios between variant and wild-type likelihoods).

To understand the knowledge transfer from sequence modeling in pLMs to the downstream variant effect prediction, we followed the protein sequence-structure-function paradigm and examined sequence and structure awareness of pLMs in association with their fitness sensitivity. Sequence awareness measures a model’s ability to recover masked amino acids, aligned with the goal of sequence modeling. Similarly we defined structure awareness as a model’s ability to predict three structural properties: secondary structure (SS), relative solvent accessibility (RSA) and $C_{\beta }$ distance map (DM). As conventional pLMs only produce distribution over amino acids, a simple multilayer perceptron (MLP) was trained for each property using amino acid embeddings at the final layer of the trained sequence models. Continuous property labels (RSA and DM) were discretized to make each task classification. The MLPs were trained over the same family-specific structure property sets, utilized to develop structure-informed pLMs in Sec. 2.3.

We pre-trained five bidirectional transformer encoder (BERT) pLMs over combinations of four architectures and two Pfam representative proteomes domain sequences. The four architecture variations of BERT were utilized where from B4 to B1 more self-attention heads, layers, and neurons in the position specific feed-forward layer are included (See details in Sec. 3.3 and architectures in Table 5). The two sequence datasets were RP15 and RP75 at at 15% and 75% co-membership thresholds, respectively. We selected 12 fitness sets from a benchmark set [11] and evaluated the five encoders’ sequence/structure awareness (towards target proteins) and fitness sensitivity (Table 1). We also fine-tuned the five encoders for each of the 12 protein families’ sequences and performed the same evaluation.

Numerical results showed strong correlation between sequence/structure awareness and fitness sensitivity. Among pre-trained models, the best fitness predictor, with the average Spearman’s ρ at 0.302, exhibited the highest sequence awareness of 0.411. We also found awareness supporting for the second best predictor, processing the highest overall sequence+structure awareness (*ASRD* value 0.485), and the third one having the best overall structure awareness (*SRD* value 0.557). After fine-tuning on family sequences, these models showed significant improvement in fitness prediction, with the average ρ increasing from ~0.25 to 0.55 and the standard deviation reducing almost by half. It is noteworthy that such improvements in fitness prediction were accompanied with those in sequence awareness (*AA* increasing from 0.4 to nearly 0.9) and structure awareness (*SRD* increasing from 0.55 to 0.60). The best fitness predictor among the fine-tuned models was also the one with the highest structure and structure awareness.

These results indicate that the amount of biological contexts learned in pLMs is correlated with their abilities for variant effect prediction, which motivates our approach to purposed injection of structure awareness in pLMs for variant effect prediction.

### 2.2 Structure information abates overfitting in sequence modeling

Although fine-tuning with family sequences is an effective way to adapt pre-trained meta pLMs for the local and fine-grained evolutionary landscapes of target proteins (as demonstrated in the average performances in Table 1), overfitting may occur and cause poor performances in predicting variant fitness, especially for over-parameterized pLMs. In fact, ESM-1v has experienced rapid overfitting when naively fine-tuned on family sequences and had to resort to a new strategy called spiked fine-tuning [19].

To examine the overfitting issue during fine-tuning, we selected the encoder *RP15 B2* (50M parameters) and examined its fitness prediction performances for each of the 12 datasets (families) before and after fine-tuning. Fig. shows analyses over the 12 families, ordered in decreasing difference in Spearman’s correlation between pre-trained (gray square) and fine-tuned (black square). We found that one third (the 4 to the right) of the cases had the overfit issue, indicated by worse fitness ranking (up to 0.1) after fine-tuning.

**Fig. A1:**
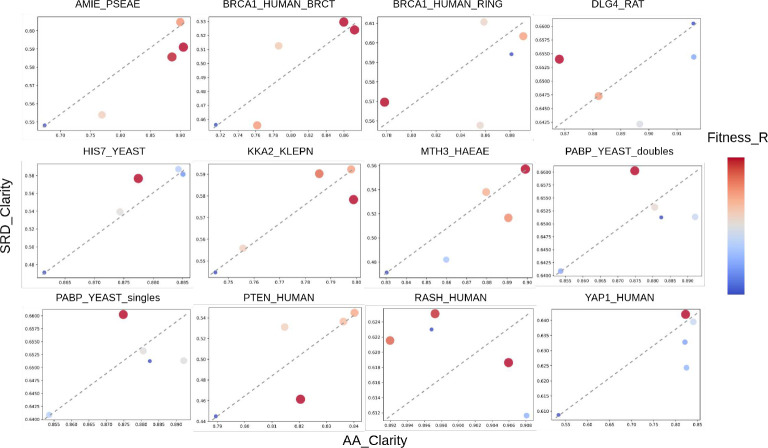
The five BERT-based models, when applied to different proteins, exhibit different trade-offs between sequence semantics and structure awareness for their sensitivity towards fitness prediction.

To provide a possible remedy to overfit, we examined the sequence and structure awareness of fine-tuned *RP15_B2* in each case in comparison to those of the other four fine-tuned models. Supplemental Fig. A1 shows that, once reaching the local landscape with fine-tuning, there might be a trade-off between sequence and structure awareness and simultaneous improvements in both may no longer be feasible. In the overfitting cases (*DLG4*, *PABP*, and *YAP1*), better fitness predictors than *RP15_B2* existed with family-specific trade-offs tilted more toward structure awareness.

These results indicate that the ability to adjust the trade-off between sequence and structure awareness during fine-tuning could mitigate the overfitting issue, which provides another motivation to our approach to controllable injection of structure awareness in pLMs for variant effect prediction.

### 2.3 Structure-informed pLMs predict variant fitness robustly

Motivated to inject structure awareness into pLM purposely (Sec. 2.1) and controllably (Sec. 2.2) for robust variant effect prediction, we propose to extend masked language modeling in pLMs and introduce structure-informed (SI)-pLMs through cross-modal masked modeling (denoising) (Sec. 3.2). This SI-pLM framework consists of two simultaneous learning tasks: intra-modal sequence denoising as in conventional pLMs and cross-modal sequence-to-structure denoising as auxiliary tasks. Specifically, the auxiliary tasks include predictions from masked sequences to three structure properties as in evaluating structure awareness: two 1D properties (relative solvent accessibility or RSA and secondary structure or SS, per residue) and one 2D property (distograms for residue pairs). Accordingly, additional decoders are appended to a transformer-based language model; and additional losses are averaged and weighted by a controllable hyperparameter λ, then added to the loss of masked language modeling, so as to train SI-pLMs. Our SI-pLMs are illustrated in Fig. 2 and detailed in Sec. 3.3. Compared to conventional sequence-only pLMs, SI-pLMs are more general and its special case when λ = 0 is equivalent to conventional pLMs.

Our most advanced version of SI-pLMs uses both sequence-structure pairs and unpaired sequences as training data and a small fitness-labeled subset as validation to tune λ over a grid 0, 0.5, 2, 20 (Sec. 3.4). We assessed its performance based on the encoder *RP15_B2* (50M parameters) over a benchmark dataset for fitness prediction. Each protein labeled with variant effects in this dataset comes with an MSA file queried over the UniRef100 database. We further curated crystal or predicted structures for each family sequence available from Protein Data Bank and AlphaFold Structure Database (AFDB), respectively. Apart from uploaded structures in AFDB, we didn’t additionally predict structures using the AlphaFold2 software, so not necessarily every sequence has a paired structure. As more than 99% sequences in viral families have no structures, they are excluded from this study. The final dataset contains 35 DMS sets (12 of which were used in the motivational studies in Sec. 2.1 and Sec. 2.2). More details about data preparation can be found in Sec. 3.4.

We compared our SI-pLM in ranking (Spearman’s ρ) and classification (AUROC and AUPRC) to 10 competing methods including alignment-based PSSM, EVMutation, DeepSequence, and MSA-transformer as well as alignment-free UniRep, WaveNet, TAPE, ESM-1b, and ESM-1v (pretrained and fine-tuned). For ranking performances we also split the families by sequence information (measured by MSA depth ($N_{\text{eff}}/L$ with cutoffs at 10 and 60). Table 2 shows that our alignment-free SI-pLM, using both structure-paired and unpaired sequences and label validation (for λ), outperformed all other alignment-based or alignment-free methods in overall ranking. As SI-pLM is fine-tuned over family sequences, its ranking performances were impacted by the level of sequence information but still among top 2 and 3 for families of medium and low MSA depth, respectively. Even for ranking in the low MSA-depth cases, it only trailed alignment-based MSA-transformer with twice amount of parameters and fine-tuned ESM-1v with 13-times more parameters. As to classification performances, SI-pLM also ranked top 2 and 3 in AUROC and AUPRC, respectively, whereas DeepSequence did the best. By comparing our SI-pLM and each other method through the Wilcoxon signed-rank test (a non-parametric version of the paired t-test), we showed that its performance gains over 7 of the 10 competing methods were statistically significant *p* < 0.05. The performance comparison for each individual variant set was reported in Fig. 4 and showed that our SI-pLM was top 1 or 2 among the five best methods for 20 of the 35 variant sets. Additional performance split over mutation depth was reported in Supplemental Table A1.

**Fig. 3:**
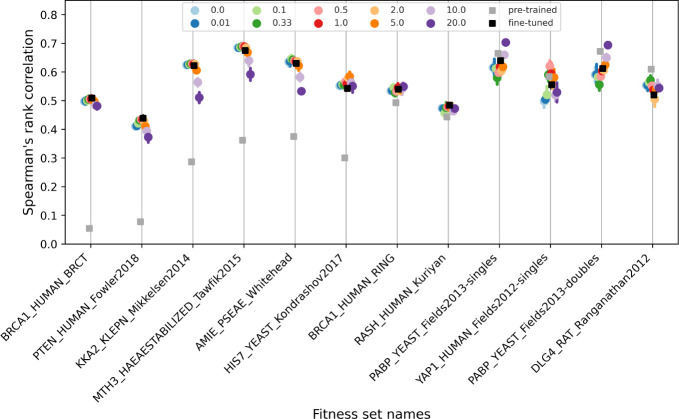
Fitness prediction performance declines after fine-tuning when overfitting happens in sequence modeling. The meta pre-trained model (gray square) was fine-tuned (black square) over family sequences to enhance evolutionary awareness specifically for the target protein. This fine-tuning process aimed to improve fitness prediction, as demonstrated by the fitness sets on the left side. However, as we moved towards the right side, we noticed diminishing improvement and even increasing deterioration in Spearman’s rank correlation, indicating the occurrence of overfitting over the sequence modeling task. Such over-finetuning or overfitting was evident in the last four sets on the right. Our initial experiments indicate that by incorporating structure information through model regularization, we can enhance the robustness and effectiveness of fine-tuning for fitness prediction. The weighting hyper-parameter λ, which balanced the contribution of structure tasks within the overall objective function, was tested over a grid of values from 0.0 to 20.0, as illustrated in the legend.

**Fig. 4:**
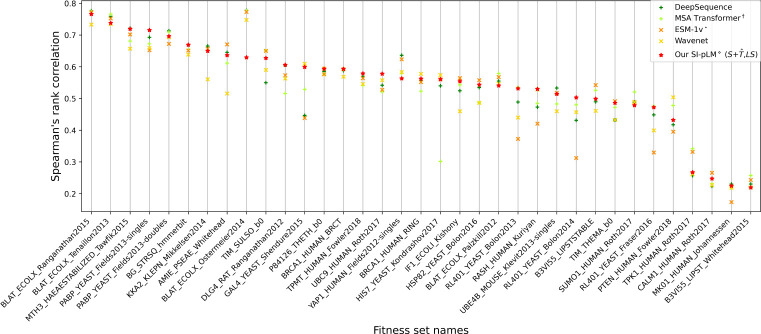
Mutation effect prediction on DMS benchmark sets

These results show that our SI-pLM, although much smaller compared to competing pLMs such as ESM-1v and MSA-transformer, has competitive performance robustly across ranking and classification tasks and across protein families of various sequence information. Furthermore, we found that it is capable of mitigating overfitting for affected families often by tuning up λ for more structure awareness (Fig. 3), which provides robustness across protein families that may or may not experience overfit in pLM fine-tuning.

### 2.4 Learned distributions in structural properties enhance variant effect scoring

Our SI-pLMs not only regularize learning the distributions in protein sequence (amino acid types), but also learn the distributions in protein structure (structural properties in this study). We assessed the potential of using the structure distributions (additionally) to rank or classify variant effects (Table 3). Using the learned distributions in structural properties *alone*, individually or together, did not rank or classify variant effects better than using the learned distributions in sequence (with structural regularization).

However, using the learned distributions in structural properties *in addition to* those in sequence did improve ranking (in Spearman’s ρ) and classification (in AUROC). Distributions in contact map (residue–residue edge features) was the best performer among single structural properties (including two residue node features) and also enhanced sequence distributions the most in ranking.

### 2.5 Ablation study

To quantify the contributions of various components of our SI-pLM, we started with the Pfam RP15 pre-trained pLM *RP15-B2* and incrementally included the following symbolized components in order:

†: sequence pre-trained only

⋆: fine-tuned over target protein’s family sequences including $\overset{ˆ}{S}$, the subset of sequences with experimental or predicted structures available, and S, the set of all sequences (training-set split).

⋄: fine-tuned over target protein’s family sequences ($\overset{ˆ}{S}$ or S) and structures ($\overset{ˆ}{T}$, the structure set corresponding to $\overset{ˆ}{S}$.

*LS*: select the model with the best zero-shot fitness ranking performances based on the label validation set.

We reported these ablated versions’ performances again in ranking and classification over the 35 DMS variant sets. We also split the ranking performances into families of low to high MSA depths ($N_{\text{eff}}/L$ cutoffs at 10 and 60) as well as low to high structure availability $\left(|\overset{ˆ}{\overset{ˆ}{S}}|/|S|\right)$ ranging from 27% to 95% with cutoffs at 50% and 90%). Table 4 shows increasing performances as model components were incrementally included, indicating the positive contribution of each. Compared to the pre-trained pLM^†^ (pfam), fine-tuning (pLM^⋆^) greatly improved ranking performances (ρ increased from 0.43 to 0.50), especially for families of low to medium MSA depth, as well as classification performances (AUROC increased from 0.75 to 0.78). Fine-tuning with more sequences (S versus $\overset{ˆ}{S}$) also helped, especially for families of low to medium MSA depth. When structure information was first introduced and paired sequences and structures ($\overset{ˆ}{S}$ and $\overset{ˆ}{T}$) were used, structure-informed SI-pLM^⋄^ $\left(\overset{ˆ}{S}+\overset{ˆ}{T}\right)$ improved significantly compared to the fine-tuned counterpart without structures (pLM^⋆^ $\left(\overset{ˆ}{S}\right)$), with overall ρ increased from 0.508 to 0.526 and AUROC increased from 0.778 to 0.794. The structure-boosted ranking improvements were more pronounced when structure availability was higher. Additionally using structure-unpaired sequences in SI-pLM^⋄^ $\left(S+\overset{ˆ}{T}\right)$ especially helped the families with low structure availability (ρ increased from 0.530 to 0.542) but can help the families with high structure availability as well. Lastly, compared to self-supervised validation loss without the need of fitness labels, using the supervised fitness validation (*LS*) further improved the overall ranking (overall ρ increased from 0.530 to 0.546, although the improvement was diminished without unpaired sequences). This result suggests that self-supervised learning (fine-tuned) and the downstream variant fitness prediction are not perfectly aligned in objectives and a small labeled dataset, if available, could help reduce the alignment gap and improving the downstream variant effect prediction. Taken together, other than fine-tuning, the biggest contributor was injecting structure awareness and another boost was from the combination of supervised label validation and unpaired sequences.

### 2.6 Understanding the impact of structure regularization on sequence embedding

Since transformer-based pLMs have exhibited capabilities of learning informative representations for proteins in terms of structures and functions [15, 34, 35], we are driven to answer the following two questions through examining the embedding manifolds in pLMs: 1) how fitness landscapes reflect in the latent embedding space of pLM, and 2) after injecting structure information, how embedding manifolds are altered relative to sequence-only ones.

**Fig. 5:**
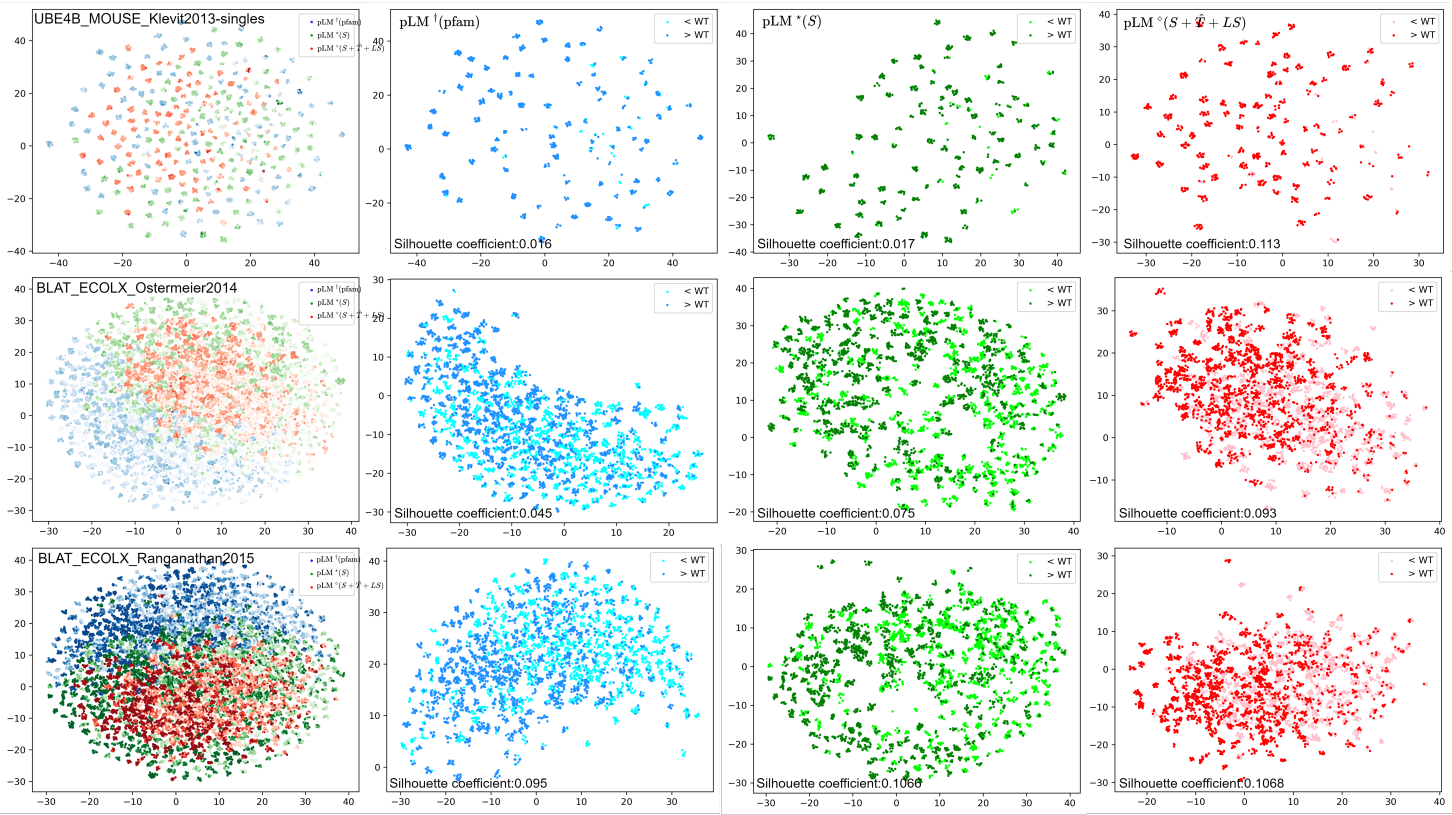
Low-dimension manifolds of variant embeddings by various pLMs (columns) for three representative proteins (rows) indicate that structure information led to better separation of high and low-fitness clusters. Whereas the first column is the union of three models: sequence pre-trained model (pLM^†^(pfam), blue), sequence fine-tuned model (pLM^⋆^(*S*), green), structure-informed model (SI-pLM^⋄$\left(S+\overset{ˆ}{T}+LS\right)$^, red), each of the last three columns corresponds to one of the three models. In each figure, each point represents a variant, is located at the averaged embedding over mutant positions, and is colored according to the experimental fitness values (darker for higher fitness, continuous in the first column and binarized relative to the wild type in the other three columns).

To answer these questions, we select three protein sets (*UBEX_MOUSE* and two *BLAT_ECOLX* studies), where SI-pLMs have obvious gains in fitness ranking and classification after injecting structure information, and probe the embedding of sequence variants at the last self-attention layer. We investigate three models: domain sequence pre-trained pLM (pLM^†^(pfam)), family sequence fine-tuned pLM (pLM^⋆^(*S*)) and structure-informed fine-tuned pLM (SI-pLM^⋄^$\left(S+\overset{ˆ}{T}+LS\right)$). The two-dimension embedding manifold generated by UMAP are shown in Fig. 5, where each row is a protein case and each column is the set of three models or an individual model. In each figure, each dot represents the position of one mutation in the embedding space and its color transparency indicates experimental fitness values (darker colors for more fit variants). As our pLMs were only trained with the residue-level task, which is denoising (or recovering) masked residues, we tried two averaging approaches to obtain the sequence-level embedding for each mutant sequence: all-position averaging and mutant-position averaging. We find that the manifold of mutant-position averaged embeddings better capture target protein’s fitness landscape (Fig. 5) relative to all-position averaged embeddings (data not shown). Our interpretation is that the small perturbation in embedding space induced by amino acid substitutions at few positions (most mutations in our DMS datasets are single-site) is largely washed out if their embeddings are averaged along with those of all other unchanged positions. Rather, averaging residue-wise embeddings only over mutated positions (as in Fig. 5) provides more sensitivity to our analyses.

**Fig. A2:**
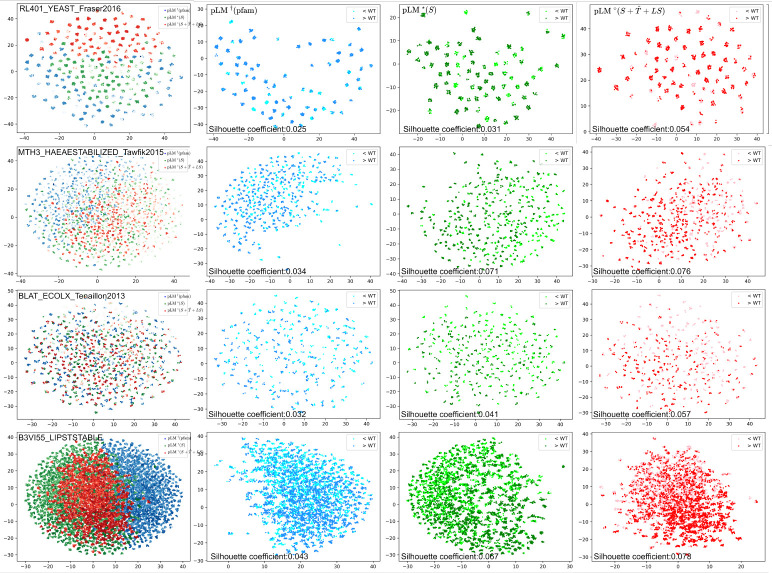
Low-dimension manifold of mutant embedding space for four proteins (correspondng to four rows). Each point represents the averaged embedding over mutated positions in one variant sequence, and the color transparency indicates experimental fitness values that darker points are more fit mutations. The original embedding dimension is 768 and, reduced to 2 by UMAP. Embeddings from outputs of the last self-attention layer are probed here, and three models (corresponding to the last 3 columns) are compared together: sequence pre-trained model (pLM^†^(pfam), blue), sequence fine-tuned model (pLM^⋆^(*S*), green), and structure-informed model (SI-pLM^⋄$\left(S+\overset{ˆ}{T}+LS\right)$^, red)

Fig. 5 shows that all three types of models lead to certain separation between sequence variants of high versus low fitness, which echoes the previously observed effectiveness of pLMs for zero-shot variant effect prediction. Fine-tuning over target family sequences or sequences & structures led to better separation, as quantified by the higher silhouette coefficients (more compactness within high/low-fitness clusters and more separation across high/low-fitness clusters). Such better separation of embedding manifolds in the latent space makes corresponding model more ready for fitness prediction. Importantly, compared to sequence-only fine-tuned models, our SI-pLMs finetuned over target family sequences and structures, had even better cluster separation with higher silhouette coefficients, making them ready to better distinguish variants of low versus high fitness values. Four more protein sets were examined in the Supplemental Fig. A2 where similar observations were made. These results demonstrate the impact of structure information as a context provider and model regularizer.

## 3 Methods

### 3.1 Preliminaries

Proteins possess multimodal attributes in the forms of 1D sequences, 2D inter-residue distograms/anglegrams and 3D structures. Amino acids act as the fundamental units, following sequential constraints to form functional proteins, which subsequently fold into particular 3D structures. In this paper, we denote protein primary sequences as $S_{\text{seq}}$ and structures as $S_{\text{struct}}$.

Sequence-based protein language modeling (pLM) estimates the likelihood of protein sequences $\left(p\left(S_{\text{seq}}\right)\right)$ with models trained over large corpora of natural sequences such as UniRef and BFD. Masked language modeling (MLM) is one of the major self-supervision frameworks for pLM. It takes a noised sequence with random positions masked and recovers amino acid types at those masked positions, thus modeling the pseudo log-likelihood (pll) of sequence: $pll\left(S_{seq}\right)=log\prod_{\left\{m\right\}}\;p(S_{seq}^{\left\{m\right\}}|S_{seq}^{\backslash \{m\}})=\sum_{\left\{m\right\}}\;log\;p(S_{seq}^{\left\{m\right\}}|S_{seq}^{\backslash \{m\}})$, where {m} denotes a set of masked positions and \{m} denotes its complement. Another pLM framework, next token prediction (NTP) recovers the whole sequence token by token through modeling exact likelihood $\left(p\left(S_{\text{seq}}\right)=\prod_{i=1}^{N}\;p\left(a_{i}|a_{1..i-1}\right)\right)$ following the causal order (left-to-right or right-to-left).

### 3.2 Cross-modal masked learning (denoising) framework

Extending beyond sequence pseudo log-likelihoods, we propose to model the following cross-modal pseudo log-likelihood (CMPL):

(1) $$\mathrm{CMPL}\left(S_{\text{seq}},S_{\text{struct}}\right)=\log p\left(S_{\text{seq}}^{\left\{m\right\}}|S_{\text{seq}}^{\backslash \{m\}}\right)+\log p\left(S_{\text{struct}}|S_{\text{seq}}^{\backslash \{m\}}\right)$$

The first term $log\;p(S_{seq}^{\left\{m\right\}}|S_{seq}^{\backslash \{m\}})$ is exactly aligned with MLM in conventional sequence-only protein language models. And the second term, which can be multiplied by a weighting hyperparameter λ, recovers protein structures from masked sequences.

We represent $S_{\text{seq}}$ by a string of amino-acid type $a_{i}$ over residue i. We used twenty nine choices for amino acid type $a_{i}$, including twenty standard amino acids, two special amino acids (U and O), three ambiguity letters (X, B, and Z), and four special tokens used in language models. And we represent $S_{\text{struct}}$ by a residue contact graph G={V,E} where nodes in V are amino acids and edges in E represent interactions among amino acids (if pairwise $C_{\beta }$ distances are within 8Å). Therefore, structures are represented by nodes and edges, in other words, structural properties. We selected secondary structure $s_{i}$ (3 classes) and relative solvent accessibility $r_{i}$ (binarized with the cutoff of 0.25) for nodes, and pairwise $C_{\beta }$ distance $d_{ij}$ for edges, which are essential attributes for protein function and can be quickly acquired from structure files.

The second term can now be factorized over nodes and edges while assuming their independence:

(2) $$log\;p(S_{\text{struct}}|S_{\text{seq}}^{\backslash \{m\}}\;\approx log(p(V^{S_{\text{struct}}}|a_{\backslash \{m\}})p(E^{S_{\text{struct}}}|a_{\backslash \{m\}}))\;\approx log\;p(V_{SS}|a_{\backslash \{m\}})+log\;p(V_{RSA}|a_{\backslash \{m\}})+log\;p(E_{\text{DistMap}}|a_{\backslash \{m\}})\;=\sum_{i=1}^{L}\;log\;p(s_{i}|a_{\backslash \{m\}})+\sum_{i=1}^{L}\;log\;p(r_{i}|a_{\backslash \{m\}})+\sum_{i=1}^{L}\;\sum_{j>i}^{L}\;log\;p(d_{ij}|a_{\backslash \{m\}}))$$

The resulting expression provides the foundation for the training losses (objective functions) in our structure-informed protein language models, which is detailed as follows.

### 3.3 Structure-informed protein language models

Next, we describe the neural network parameterization for the CMPL framework and objective functions to train the model, as well as the variant scoring method under zero-shot transfer setting.

#### 3.3.1 Model architecture

The diagram of our model is shown in Fig. 2. Built upon a BERT-based protein LM, we developed simple decoders to predict variables in two modalities employing amino acid embeddings and attention scores from pLM. We accommodated the dimensionality requirement in the 2D contact-map prediction with a outer product module.

##### Protein LM

We applied a BERT-based architecture and pre-trained the model over domain sequences from Pfam. BERT makes use of the transformer encoder, an attention mechanism that learns contextual relations between entities in input data. To be more specific on protein sequence inputs, attention enables each amino acid selectively attending to other positions for tailored information aggregation to fulfil prediction tasks. Since attentions are conducted within positions of a single protein, this is further called self-attention, to be distinguished from cross-attention. Multiple self-attention blocks (termed heads) are utilized to assemble a self-attention layer, and multiple self-attention layers forming the whole model. Position-wise feed-forward module is attached after multi-head self-attention module in each layer to let embeddings being updated before entering the next layer. At layer i, the multi-head self-attention is:

(3) $$\text{MultiHeadSelfAttention}\left(X_{q},X_{k},X_{v}\right)\;=\left[{\text{head}}_{1}\left|\ldots \right|{\text{head}}_{H}\right]|W^{o}\;\;{\text{where}\;\text{head}}_{h}\;=\text{Attention}\left(X_{q}W_{h}^{q},X_{k}W_{h}^{k},X_{v}W_{h}^{v}\right)\;\;\;=\text{softmax}\left(\frac{\left(X_{q}W_{h}^{q}\right){\left(X_{k}W_{h}^{k}\right)}^{T}}{\sqrt{d_{k}}}\right)\left(X_{v}W_{h}^{v}\right)$$

where $X_{q}=X_{k}=X_{v}=X_{i}\in R^{L\times s}$ in self-attention, and [.|⋅] is a concatenation operation. $W_{h}^{q},W_{h}^{k}\in R^{s\times d_{k}/H},W_{h}^{v}\in R^{s\times d_{v}/H}$ are weight matrices to transform input features X into query, key and value matrices. $W^{o}\in R^{d_{v}\times s}$ linearly transforms concatenated output features from multi-head self-attention. An ”add & norm” operation is applied afterwards $X_{a}=LayerNorm(MultiHeadSelfAttention\left.\left(X_{q},X_{k},X_{v}\right)+X;\gamma_{a},\beta_{a}\right)$. The position-wise feed-forward module is:

(4) $$X_{f}\;=GELU\left(X_{a}W_{1}\right)W_{2}\;X_{i+1}\;=LN\left(X_{f}+X_{s};\gamma_{f},\beta_{f}\right)$$

The Gaussian Error Linear Units (GELU) [36] activation function and layer normalization (LN) [37] are used to reduce overfitting.

We have tested five BERT-based encoders with increasing training data and model complexity as follows:

##### Outer product module

In order to apply amino acid features from pLM for 2D contact-map prediction, we designed an outer product module to convert positional embeddings to pairwise ones. for any pair of amino acid i,j and their embeddings from the last self-attention layer in pLM $x_{i}^{L},x_{j}^{L}\in R^{s}$, the embedding dimension is firstly reduced for left and right vectors, then take the outer product: $X_{ij}=x_{i}^{L}W_{l}⊗x_{j}^{L}W_{r}$, where $W_{l},W_{r}\in R^{s\times d_{m}}$ are left and right linear transformation matrices. The outer product matrix ${X_{ij}\in R}^{d_{m}\times d_{m}}$ is then flattened and linearly transformed: $x_{ij}^{\text{pair}}=flatten\;\left(X_{ij}\right)W_{s}$, where $W_{s}\in R^{{\left(d_{m}\right)}^{2}\times d_{s}},x_{ij}^{\text{pair}}\in R^{d_{s}}$. This pairwise feature map is later concatenated with attention matrices from all self-attention heads along the feature dimension.

##### Label prediction heads

Since transformer encoder-based protein language models have been demonstrated effective to learn informative protein representations, we only considered very simple decoders to predict sequence and structure self-supervision labels. The positional feature $\left({\tilde{X}}^{\text{pos}}\in R^{L\times s}\right)$ or pairwise feature ${\tilde{X}}^{\text{pair}}\in R^{L\times L\times \left(d_{a}+d_{s}\right)}$ pass through individual two-layer MLPs to generate class logits for each label: $\overset{ˆ}{y}=LN\left(GELU\left(\tilde{X}W_{1}^{D}\right)\right)W_{2}^{D}$, where sizes of $W_{1}^{D}$ and $W_{2}^{D}$ are label-dependent, and $\overset{ˆ}{y}\in R^{C},\;C$ as number of classes for each label. We note that pairwise feature map is symmetrized $\left({\tilde{X}}^{\text{pair}}=\frac{1}{2}(X^{\text{pair}}+X^{{\text{pair}}^{⊤}})\right)$ before feed into the MLP, relieving the model from learning the symmetry in contact-maps.

##### 3.3.2 Training objectives

The softmax function is applied on class logits to generate a probability distribution p over classes. Following Eq. 1 and 2, the overall training objective is $ℒ={ℒ}_{aa}+\lambda \left({ℒ}_{ss}+\right.\left.{ℒ}_{rsa}+{ℒ}_{cm}\right)$, where λ is the hyper-parameter regulating the strength of structure tasks.


(5) $${ℒ}_{aa}\;=E_{D^{seq}}\left[\frac{1}{\left|\{m\}\right|}\sum_{\overset{ˆ}{a}\in a_{\left\{m\right\}}}\;-log\left(p_{aa}(\overset{ˆ}{a})\right)\right]\;{ℒ}_{ss}\;=E_{D^{\mathrm{struct}}}\left[\frac{1}{L}\sum_{i=1}^{L}\;-log\left(p_{\mathrm{ss}}\left(s_{i}\right)\right)\right]\;{ℒ}_{\mathrm{rsa}}\;=E_{D^{\mathrm{struct}}}\left[\frac{1}{L}\sum_{i=1}^{L}\;-log\left(p_{\mathrm{rsa}}\left(r_{i}\right)\right)\right]\;{ℒ}_{\mathrm{cm}}\;=E_{D_{\mathrm{struct}}}\left[\frac{1}{\left|\{ij\}\right|}\sum_{i=1}^{L}\;\sum_{j>i}^{L}\;-log\left(p_{cm}\left(d_{ij}\right)\right)\right]$$

As each term is calculated over randomly sampled data points in each batch, there may be batches which only contain structure-absent sequences when augmented sequence data is used. For these cases where size of $D^{\mathrm{struct}}$ is zero, all three structure-property loss terms are set to 0 in such a batch.

#### 3.3.3 Variant effect scoring

Sequence-based methods is built on the modeling goal that a well trained network over natural sequences in target MSA should learn evolutionary constraints among residues underpinning function-favored sequences. In other words, compared to the probability that a model assigns to the wild-type amino acid, higher probability should be given to amino acids with better functional propensity than wild-types and vice versa. We mask all variant positions and score each single-site variant (M={i}) by the log odds between the variant and wild-type amino acids, which is equivalent to $log\frac{p\left(a_{i}^{mut}|a_{\backslash M}\right)}{p\left(a_{i}^{wt}|a_{\backslash M}\right)}$.

For multi-site mutations (|M|>1), the additive assumption is used and the above score is summed over i∈M.

Our structure-informed pLMs provide more options to score variant effects. First, we could use the same expression of log odds in amino-acid (AA) types but note that the learned odds are regularized by structural information. Second, we could use additional log odds in structural properties, including secondary structure (SS) classes, relative solvent accessibility (RSA) classes, and contact-map (CM) distance bins, or the sum of the log odds in all three structural properties. We also consider the log odds not only for the mutation site but for its local environment (any residues within 8Å in $C_{\beta }$ distances. Lastly, we could use the sum of log odds in both amino acid types (sequence) and structural properties.

### 3.4 Datasets

#### Sequence dataset

The sequence data to pre-train pLMs are non-redundant domain sequences of representative proteomes (RP) downloaded from Pfam. Representative proteomes are groupings of similar proteomes, whereby a single proteome is chosen to best represent the set of grouped proteomes in terms of both sequence and annotation information [38]. The grouping redundancy is controlled by the co-membership threshold (at four levels, 75, 55, 35 and 15%) that lower value produces larger groupings, hence resulting in less redundant sequence sets. We used the RP15 set as pretraining corpus since it is the most cost-efficient one. We also trained a version of our largest pLM with the RP75 set. The alignments were removed from original downloaded MSA files to train on primary sequences.

The family fine-tuning sequence dataset was downloaded from Wavenet [13]. Each protein comes with a MSA file containing homologous sequences queried from UniRef100 database. An identity-based weighting score is attached to each sequence that sequences with lower identity among homolog population have higher values. We direct readers to DeepSequence [11] for details of weight calculation. The fine-tuning was also conducted over unaligned primary sequences. Different from pre-training, training samples were re-weighted according to their weighting scores.

#### Structure dataset

As we are dealing with protein domain sequences, a Uniprot accession number together with a pair of start and end indices can uniquely define one sequence data in our dataset. We queried RCSB Protein Data Bank (PDB) and AlphaFold Protein Structure Database (AlphaFold DB) for available structures to family sequences. To collect crystal structures from RCSB PDB, we first curated a set of 100% non-redundant protein polymer entities for Uniprot accession number candidate set. As one protein polymer entity may have multiple instances (e.g. chains in homopolymer proteins), we kept the polymer instance with the longest coverage and least unobserved residues. At this point, we ended up with a set of structure instances with no identical sequences to each other. Then we excluded structures having no overlapping with target sequence by inspecting start and end indices. This pipeline was conducted over each family to collect its non-redundant structure dataset. We note that partial sequences have no structure data since we didn’t further run AlphaFold2 inference if no structures can be found on AFDB. The structure files in mmCIF format were downloaded for calculation of secondary structure and relative solvent accessibility using Biopython’s DSSP module. $C_{\beta }$ distance-maps were manually generated and transformed to contact-maps with the cutoff of 8*Å*.

#### Mutation effect datasets

The wildly used mutation fitness benchmark set was downloaded from DeepSequence [11] with one tRNA set excluded as we are handling protein mutations. We only considered missense mutations in all experiments. Since structure availability is extremely low over viral families, fitness sets of viral proteins were also not considered in this study. The predicted mutation fitness scores of all competing methods we compared to were acquired from ESM-1v’s github repo.

To further mitigate the discrepancy between self-supervision tasks and fitness prediction, we hope to directly use label information to select the most fitness-sensitive model for inference. A small yet representative mutation subset is selected that one mutation, including multi-site mutations, is randomly picked at each mutant position without replacement.

### 3.5 Competing methods

We focus on the main sequence modeling approaches described in the introduction, including a number of protein-specific methods: PSSM (site-independent) model, EVmutation (Potts model) [10], DeepSequence [11], which were all trained on aligned sequences, and Wavenet [13], trained over unaligned sequences with autoregressive generative models. We also include representative protein language models trained across protein families that leverage alignments during training, such as the MSA Transformer [18] or that are alignment-free, such as UniRep [7], TAPE [8], ESM-1b [15], ESM-1v [19] and ProtBert-BFD [17]. Our SI-pLM is alignment-free with additional structure modeling (see comparison with other pLMs as follows).

## 4 Conclusion

In this study, we take the perspective of assessing and injecting structural contexts into protein language models toward variant effect predictors. We found that, although commonly-used sequence fine-tuning may improve sequence and structure awareness toward better variant effect prediction, over-finetuning could occur; and the balance between sequence and structure awareness needs to be purposeful and controllable.

Extending the sequence-only masked language modeling, we introduce a framework of cross-modality masked learning for purposeful and controllable injection of structure awareness into protein language models. This framework is agnostic to protein language models (pLMs) in the sense that it can modify the architecture of any existing transformer-based pLMs with structural decoders and the training losses of any existing transformer-based pLMs through auxiliary sequence-to-structure denoising tasks. This framework does not demand protein structure data as additional inputs to protein sequences during inference (and variant effect prediction), while utilizing both sequences paired with available structures and unpaired sequences without structures (no multiple sequence alignments are needed either).

Numerical results over benchmarks for variant effect prediction indicate that, whereas our SI-pLMs are compact in model size compared to competing language models, they are consistently top performers regardless of the protein family being evolutionary information-rich/poor or being prone to over-finetuning. Learned distributions in structural contexts not only regularize those in sequence but could also enhance the latter’s variant scoring performances. Ablation studies revealed major contributors of the numerical performances, whereas visualization of the latent embeddings showed that structure information led to better separation of low/high-fitness sequence clusters and better readiness for zero-shot variant effect prediction.

## Figures and Tables

**Table 1:** Five in-house pLMs’ variant fitness sensitivity (Spearman’s rank correlation between model scoring and experiment fitness measurements) versus the awareness of target protein’s sequence and structure (clarity scores detailed next) evaluated over 12 fitness datasets. The normalized clarity score, derived from perplexity, represents models’ awareness towards sequences or structures of target proteins. Integers in the parenthesis are class numbers for corresponding properties. *SRD*, as a summarized score for structure awareness, takes geometric mean of Secondary Structure(SS), Relative Solvent Accessibility(RSA) and Distance Map(DM). *ASRD* additionally includes sequence awareness on Amino Acids(AA) in averaging. All values are reported in percentage format and values with smaller font sizes are standard deviations for the preceding quantities. Superscripts refer to ranking among the group and the maximum clarity values are underscored for each property.

Encoder	Spearman’s ρ ↑ (×1e-2)		Normalized Clarity(×1e-2) on WT proteins ↑											

			AA(20)		SS(3)		RSA(2)		DM(32)		SRD		ASRD	

Sequence-Pretrained Meta pLMs														

RP75_B1	18.97	14.38	28.78	22.40	55.98	8.23	35.34	7.91	73.82	18.08	52.52	10.46	43.03	13.51
RP15_B1	**24.89** ^3^	14.83	35.92	25.03	58.37	5.82	37.93	6.25	78.22	14.38	55.66	8.12	47.79	13.02
RP15_B2	**25.91** ^2^	16.40	38.40	24.89	58.89	5.67	37.32	6.21	76.73	15.84	55.13	8.34	48.47	13.29
RP15_B3	**30.23** ^1^	18.10	41.14	29.12	56.25	7.63	35.72	7.90	71.68	19.10	52.25	10.61	46.66	16.97
RP15_B4	24.03	17.32	36.28	26.65	55.87	7.78	35.32	7.80	71.24	18.66	51.85	10.51	45.30	15.98

Sequence-Finetuned Family-specific pLMs														

RP75_B1	55.12	10.23	83.83	10.16	61.31	3.33	41.24	3.38	80.18	9.23	58.67	4.25	63.99	3.92
RP15_B1	**55.79** ^1^	9.58	87.26	3.61	61.86	2.70	42.08	3.13	81.84	8.93	59.67	4.06	65.59	3.62
RP15_B2	55.49	8.28	85.65	4.14	61.83	2.70	41.53	3.82	80.72	9.68	59.11	4.58	64.83	4.28
RP15_B3	55.54	7.60	83.93	5.29	59.41	4.45	39.70	4.68	75.02	15.01	55.98	6.88	61.88	6.32
RP15_B4	55.24	8.07	80.46	6.78	58.83	5.35	38.62	5.86	74.95	15.27	55.30	7.93	60.64	7.23

**Table 2:** Spearman’s rank correlation, AUROC and AUPRC between model scores and experimental measurements over the fitness benchmark set. Number of parameters is also shown for each model. The top three models in each column are boldfaced with ranks in superscripts.

Model type	Model name	Spearman’s ρ by MSA depth ↑				auroc ↑	auprc ↑	p-value	#params
		Low	Medium	High	All	All	All		

Align.-based	PSSM (site-indep)	.382	.453	.449	.442	.752	.752	<1e-6	-
	EVMutation (Potts)	.445	.514	.520	.505	.782	.798	3e-6	-
	DeepSequence	.459	**.547** ^3^	**.558** ^3^	**.537** ^2^	**.804** ^1^	**.815** ^1^	0.055	-
	MSA Transformer^†^	**.479** ^1^	.539	**.562** ^2^	**.535** ^3^	**.801** ^2^	**.811** ^2^	0.136	100M

Align.-free	UniRep^†^	−.111	−.122	−.202	−.139	.414	.512	<1e-6	18.2M
	WaveNet	.460	.527	.551	.523	.792	.805	0.0008	-
	TAPE^†^	.101	.219	.018	.156	.587	.629	<1e-6	38M
	ESM-1b^†^	.431	.506	.457	.484	.771	.784	2.1e-5	650M
	ESM-1v^†^	.433	.539	.481	.511	.788	.799	0.004	650M
	ProtBert-BFD^†^	.378	.479	.487	.466	.763	.777	<1e-6	420M
	ESM-1v⋆	**.467** ^2^	**.556** ^1^	.497	.530	.797	**.809** ^3^	0.188	650M
	Our SI-pLM^⋄^	**.465** ^3^	**.550** ^2^	**.586** ^1^	**.546** ^1^	**.801** ^2^	**.809** ^3^	-	51M

† : sequence pre-trained;

⋆ : fine-tuned over target protein’s family sequences;

⋄ : fine-tuned over target protein’s family sequences and structures (if available).

**Table 3:** Comparison among sequence-based, structure-based and hybrid scoring, using learned log odds in corresponding variable(s), for variant effects in Spearman’s ρ, AUROC, and AUPRC. Compared to conventional pLMs that only use learned distributions in sequence (amino acids or AA), our structure-informed pLMs here could use learned distributions in sequence, structural properties (secondary structures or SS, relative solvent accessibility or RSA, and contact map or CM), and both. Whereas only mutation positions are considered by default, versions with subscripts ‘env’ use all neighbor positions forming local environment of mutant positions. Boldfaced are the best performances.

Type	Variable(s)	Spearman’s ρ ↑	auroc ↑	auprc ↑

Sequence	AA	.546	.800	**.806**

Structure (single property)	SS	.090	.540	.615
	SS_env_	.095	.551	.609
	RSA	.081	.545	.611
	RSA_env_	.084	.546	.599
	CM	.169	.592	.599

Structure (multi)	SS+RSA+CM	.158	.587	.590
	SS_env_+RSA_env_+CM	.144	.574	.591

Sequence + Structure	AA+SS+RSA+CM	.552	**.803**	.792
	AA+CM	**.556**	.802	.794
	AA+SS_env_+RSA_env_+CM	.554	.799	.791

**Table 4:** Ablation study of in-house pLMs from pretrained meta model, family sequence fine-tuned models to structure-informed fine-tuned models. Spearman’s rank correlation coefficient at different MSA depth are reported along with overall AUROC and AUPRC values.

Model config.	Spearman’s ρ ↑							auroc ↑	auroc ↑
	Split by MSA depth			Split by struct. %			All	All	All
	Low	Medium	High	Low	Medium	High

Our pLM^†^ (pfam)	.262	.433	.539	.359	.442	.516	.433	.747	.747
Our pLM⋆ $\left(\overset{ˆ}{S}\right)$	.435	.506	.505	.518	.492	.474	.496	.778	.791
Our pLM⋆ (S)	.453	.524	.497	.546	.507	.456	.508	.785	.798
Our SI-pLM⋄ $\left(\overset{ˆ}{S}+\overset{ˆ}{T}\right)$	.445	.535	.551	.530	.523	.528	.526	.794	.803
Our SI-pLM⋄ $\left(S+\overset{ˆ}{T}\right)$	.459	.541	.545	.542	.518	.545	.530	.794	.800
Our SI-pLM⋄ $\left(\overset{ˆ}{S}+\overset{ˆ}{T}+LS\right)$	.435	.541	.566	.529	.527	.548	.531	.801	.806
Our SI-pLM⋄ $\left(S+\overset{ˆ}{T}+LS\right)$	**.465**	**.550**	**.586**	**.553**	**.533**	**571**	**.546**	**.801**	**.809**

**Table 5:** Architecture differences among five BERT-based encoders

Encoder name	Pre-training Database (# examples)	# Layers	# Heads	Self-atten. hidden	Feed-forward hidden

RP15_B4	pfam_rp15 (12M)	4	8	768	1024
RP15_B3	…	4	8	768	3072
RP15_B2	…	6	12	768	3072
RP15_B1	…	12	12	768	3072
RP75_B1	pfam_rp75 (68M)	12	12	768	3072

**Table 6:** Comparison between our protein language models and existing representative ones

Model name	Network (# params)	Input	Pre-training Database (# examples)	Tasks

UniRep	multiplicative LSTM (18.2M)	Sequence	UniRef50 (24M)	NTP
TAPE	Transformer (38M) _ResNet; LSTM_	Sequence	Pfam (32M)	NTP, MLM
ESM-1b	Transformer (650M)	Sequence	UniRef50_2018_03 (27M)	MLM
ProtBERT-BFD	Transformer (420M)	Sequence	BFD (2122M)	MLM
MSA-transformer	Axial transformer (100M)	MSA	UniRef50_2018_03 (26M MSAs)	MLM
ESM-1v	Transformer (650M)	Sequence	UniRef90_2020_03 (98M)	MLM
Tranception	k-mer Transformer (700M)	Sequence	UniRef100 (249M)	NTP
Our pLM	Transformer (B1:92M B2:50M B3:36M B4:23M)	Sequence	Pfam_rp15 (12M); Pfam_rp75 (68M)	MLM

## Appendix A Supplemental results

### A.2 Results grouped by mutation depth

**Table A1:** Spearman’s rank correlation, AUROC and AUPRC between model scores and experimental measurements grouped by mutation depth (1 to 28) for HIS7_YEAST. Top-3 performances are with the rank in superscripts 1—3.

Model type	Model name	1	Spearman’s ρ by mutation depth ↑						All	auroc ↑	auprc ↑
			2	3	4	5	6–10	10+		All	All

Align based models	PSSM (site-indep)	.059	.197	.295	.349	.375	.448	.642	.460	.759	.841
	EVMutation (Potts)	.084	.187	.331	.414	.481	**.598** ^1^	.752	**.586** ^1^	**.8418** ^2^	**.9121** ^2^
	DeepSequence	.116	.160	.293	.367	.421	.537	**.760** ^3^	.540	.810	.888
	WaveNet	.109	.208	**.377** ^2^	**.446** ^2^	**.493** ^2^	.567	.748	.5737	.832	.908
	MSA Transformer^†^	.111	**.222** ^2^	.365	.432	.468	.394	.505	.302	.685	.811

Non-align based models	UniRep^†^	−.055	.062	.020	.002	−.047	−.205	−.190	−.182	.382	.586
	TAPE^†^	.083	.013	.027	.006	.005	.110	.410	.101	.561	.718
	ESM-1b^†^	.026	.209	.328	.380	.398	.486	.750	.500	.788	.888
	ESM-1v^†^	.096	.206	.323	.380	.417	.518	**.777** ^1^	.529	.807	.896
	ProtBert-BFD^†^	.046	.194	.300	.378	.435	.554	.752	.553	.811	.892
	ESM-1v⋆	.084	**.220** ^3^	.350	.417	.457	.554	**.769** ^2^	.560	.832	**.910** ^3^
	Our pLM^†^	**.147** ^1^	.160	.231	.271	.296	.381	.641	.391	.717	.824
	Our pLM⋆ $\left(\overset{ˆ}{S}\right)$	.102	.210	.368	**.439** ^3^	**.482** ^3^	.531	.675	.541	.823	.903
	Our pLM⋆ (*S*)	**.135** ^2^	.218	**.379** ^1^	**.457** ^1^	**.509** ^1^	.571	.665	**.5744** ^3^	**.8422** ^1^	**.9125** ^1^
	Our SI-pLM⋄ $\left(\overset{ˆ}{S}+\overset{ˆ}{T}\right)$	.111	.216	.352	.422	.471	**.577** ^3^	.674	.5736	**.840** ^3^	.908
	Our Si-pLM⋄ $\left(S+\overset{ˆ}{T}\right)$	**.127** ^3^	.219	.357	.432	.480	**.581** ^2^	.698	**.580** ^2^	.838	.909
	Our SI-pLM⋄ $\left(\overset{ˆ}{S}+\overset{ˆ}{T},LS\right)$	.104	**.248** ^1^	**.369** ^3^	.432	.464	.559	.720	.561	.836	.908
